# A people‐centred health system must be the foundation for person‐centred care in the HIV response

**DOI:** 10.1002/jia2.26125

**Published:** 2023-07-06

**Authors:** Jeffrey V. Lazarus, Rena Janamnuaysook, Georgina Caswell

**Affiliations:** ^1^ CUNY Graduate School of Public Health and Health Policy (CUNY SPH) New York New York USA; ^2^ Barcelona Institute of Global Health (ISGlobal) Hospital Clínic University of Barcelona Barcelona Spain; ^3^ Faculty of Medicine and Health Sciences University of Barcelona Barcelona Spain; ^4^ Institute of HIV Research and Innovation Bangkok Thailand; ^5^ Center of Excellence in Transgender Health Chulalongkorn University Bangkok Thailand; ^6^ Global Network of People Living with HIV Cape Town South Africa

The HIV field has been a champion in progressing global thought, action and capacity‐building towards models of healthcare that centre the lived experience, needs and preferences of affected individuals and communities. In so doing, we have markedly transitioned to an understanding that it is people in their entirety that we are concerned with, not solely their physical health or viral disease. Advocacy and activism, led by people living with HIV and key populations most at risk of and affected by HIV (e.g. men who have sex with men, people who use drugs, transgender people, sex workers, migrants, people in prisons, adolescent girls and young women in settings where HIV is highly prevalent), have pushed for the meaningful involvement of people most at risk of and impacted by HIV in all areas and stages of the HIV response [1]. This has promoted the building and strengthening of trust and mutually beneficial relationships between people living with HIV and key populations with a range of stakeholders, including clinicians, researchers and policymakers.

However, despite areas of progress, it is undeniable that person‐centred care models that serve people living with HIV and key populations remain scarce and haphazardly implemented globally. Moreover, elevating these models towards truly people‐centred health systems (PCHS) will require additional effort.

PCHS are programmes of care that provide individuals, families and communities with humanistic, holistic and trusted healthcare [2]. Notably, in such systems, healthcare must be acceptable and responsive to the needs, rights and preferences of people living with HIV and key populations [3]. Some key elements and ideas delineating PCHS include: (1) ensuring that clients and communities have a say in and the authority to make decisions regarding their health; (2) that HIV‐related services are designed according to the needs and preferences of individuals, are demand‐driven and founded on quality, safety and long‐term care engagement; (3) recognition that health systems are social institutions which thrive on mutual trust, dialogue and reciprocity; and (4) that values around justice, rights, respect and equity are at the forefront of care delivery. Furthermore, PCHS recognize that person‐centred care delivery is not limited to clinical settings and should be embraced across community settings through collaborative and coordinated approaches and partnerships [4].

Such a vision for care systems is not new. However, the need for such a vision to be systematically adopted, particularly to serve people living with HIV and key populations, has never been greater. With substantial advancements in prevention and treatment regimes, people living with HIV are living much longer. However, treatment requires a long‐term commitment to adherence by clients [5], which remains exceptionally challenging given the numerous barriers which continue to exist for people living with HIV and key populations [6, 7, 8]. These barriers include psychosocial challenges, increased prevalence of comorbidities and multimorbidities, systems and policies built around systemic stigma and discrimination and the administrative burden of navigating health systems.

Putting people at the centre of care provides an opportunity to address the multifaceted and intersectional challenges that people living with HIV and key populations encounter in a context‐adapted, sustainable and meaningful way [9]. In so doing, we create the potential to empower clients and communities and establish models of care built around shared‐decision making and HIV self‐management—all of which are essential facilitators to long‐term treatment and care adherence [10, 11]. Additionally, while decades of work have ensured that people living with HIV are living *longer*, PCHS can transition our practices to ensure that people living with HIV are living *well* [12].

In this supplement of *JIAS*, we asked nine research groups to share developments around implementing the components of PCHS in their unique settings, in countries from across Africa (including the Democratic Republic of Congo, Eswatini, Kenya, South Africa, Uganda and Zambia), North America and Europe. In this Editorial, we summarize four key themes that emerge from this supplement and share how they add to our understanding and capacity to design and implement PCHS for people living with and at risk of HIV:

(1) PCHS require meaningful and sustained engagement between stakeholders, co‐designed approaches and feedback mechanisms: at the very least, any successful effort towards the development or implementation of a PCHS requires engagement among stakeholders. When healthcare professionals listen to the voices of clients and communities, they are introduced to a perspective they may not have been aware of. Notably, engaging people and listening to their recommendations is among the first steps towards truly empowering people and facilitating the development of PCHS.

Tordoff et al. [13], for example, shed light on just how powerful and transformative a collaborative experience can be. Through community engagement activities held in the United States, they realized that the potential harm of publishing their work (e.g. increasing stigma and distrust, alongside increasing barriers to HIV prevention and treatment access) outweighed the potential benefits and, as such, decided to stop the publication of their work.

To note, stakeholders are not just clients and healthcare professionals working within a particular organization. To establish a strong and successful PCHS, partnerships between health ministers and governmental agencies, non‐governmental organizations, international donors and for‐profit private organizations are all important. This was noted by Goldstein et al. [14] in their experience in Eswatini and South Africa, where through these public−private partnerships, they were able to develop scalable programmes for HIV and non‐communicable diseases integration without requiring out‐of‐pocket costs to clients.

Importantly, to know if you are on the right track, monitoring people's experience of health systems and the care they received is essential, as was the case with Tendo‐Bugondo et al.’s [15] experience in the Democratic Republic of Congo, where they co‐designed an electronic client feedback tool. In this way they were able to pinpoint key challenges that their clients were facing, including wait times, stigma, service confidentiality and viral load turn‐around time. Such co‐designed and collaborative monitoring systems allow for the identification of gaps in quality of care and facilitate the development of realistic opportunities for system improvement.

(2) PCHS result in higher retention in care and better HIV outcomes for clients: PCHS principles require that client and community needs, rights and preferences are put first. As such, rather than choosing prevention and treatment regimens based on what is convenient or acceptable for health systems, choices and decisions should be given to clients and communities. This would serve as a way to support active and long‐term engagement.

To demonstrate the importance of this, Kabami et al. [16] developed and implemented a patient‐centred model which offered a dynamic choice in HIV biomedical prevention across distinct settings in rural Kenya and Uganda. They found that prevention products, product delivery and HIV testing modality varied between locations and over time. Notably, they report that their approach was both responsive to client preferences and resulted in higher retention in prevention services than previously reported. Nkolo et al. [17] furthered this evidence by demonstrating the importance of responding to client preferences in Uganda. They were able to determine that those who were on their preferred differentiated antiretroviral therapy model were less likely to miss appointments and achieved higher viral suppression. With these quantitative findings in sight, and recognizing that retention in care is the key to HIV prevention and treatment success in our current era of HIV care, it becomes challenging to deny the paramount importance of providing person‐centred care.

(3) Health providers encounter barriers to implementing person‐centred care: as we have seen, work towards developing and integrating person‐centred care models has been done across all aspects of the HIV response (i.e. prevention to long‐term adherence). However, the move towards integrated PCHS requires a systemic shift which addresses inequity in all its forms and for all stakeholders involved.

Mukamba et al. [18] provide interesting insight into client‐provider encounters through quantitatively parsing and characterizing patterns of person‐centred communication behaviours across Ministry of Health facilities in Zambia. They found that the majority of medical encounters (69%) were considered to have minimal to low person‐centred interactions, whereas only 8% of all medical encounters were identified as highly person‐centred. Clearly, something is amiss. Mwamba et al. [19] built on this investigation within the Zambia context by conducting focus groups with healthcare workers. Their findings suggested that while healthcare workers identify with and support principles of person‐centred care, barriers to deliver such an approach to care exist, including a work culture characterized by differential power dynamics, restricted healthcare worker autonomy, high client volume and limited human resources, laboratory capacity, infrastructure and skills to translate clients’ perspectives into practice.

To transition to PHCS, we must challenge the status quo, reflect on what it means to provide quality care and actively go against decades of ingrained policies and practices to develop new ways of working well in health systems. To facilitate this shift from traditional models to PCHS, Phillips et al. [20] provide several recommendations that resonate across global contexts. These include changes at the level of principal, policy and practice, alongside investment in and capacity‐building of healthcare providers to strengthen their engagement and enable them to build trusting relationships with clients.

(4) PCHS must go beyond focusing on acuity and instead champion wellbeing: HIV has successfully transitioned from a life‐limiting condition to a manageable chronic disease and this is largely due to major international efforts to focus on ensuring that people living with HIV everywhere, in all their diversity, can achieve viral suppression. Now, we must shift our thinking, frameworks and policies to reflect the new challenges we are met with in this current era of HIV care and management. These include: barriers to treatment adherence and retention in care, particularly for the most marginalized individuals living with HIV; increased prevalence of comorbidities and multimorbidity in people living with HIV; ageing with HIV; measuring and monitoring health‐related quality‐of‐life through the integration of client reported outcome measures; and continued efforts to eliminate HIV‐related stigma, discrimination and other human rights violations. In line with this thinking, Lazarus et al. [21] present a consensus piece developed with over 60 HIV organizations and experts from the HIV Outcomes Initiative, in which they present policy asks and recommendations for European health systems and authorities. Most notably, it is recognized that the integration of these policy innovations can support the grounds for transitioning to PCHS.

In summary, the articles in this supplement contribute to a growing evidence base demonstrating the need to adopt PCHS globally, while also sharing challenges to and strategies for implementing such models. Though it is recognized that each country is unique, and has distinct contexts and health systems in place, it is undisputed that PCHS is the foundation to addressing the challenges that exist in our current era of HIV care and management. Furthermore, the articles in this supplement serve to highlight our demonstrated capacity to incorporate human rights principles in health systems as part of the HIV response. PCHS is, therefore, not an ideal and impractical utopian concept—it is the embodiment of the right to health. Furthermore, despite the plethora of institutionalized barriers that have markedly halted global progress for years, this work shows that we *can* and *are* working towards more equal, just and trusted health systems, centred around people, their families and communities. With these learnings in mind, it is time for the HIV field to, once again, raise its banners as a champion towards equity in healthcare and strive for the accelerated and universal shift towards PCHS globally.

## COMPETING INTERESTS

No funding was received for this work. JVL reports grants and speaker fees from AbbVie, Gilead Sciences, MSD and Roche Diagnostics to his institution, speaker fees from Intercept, Janssen, Novo Nordsik and ViiV and consulting fess from Novavax, outside of the submitted work. RJ reports speaker fees from Gilead Sciences and ViiV, outside of the submitted work. GC has no competing interests to report.

## AUTHORS’ CONTRIBUTIONS

JVL drafted the editorial with input from GC and RJ. All authors further revised the editorial and approved the final, submitted version.
